# Perforator identification for propeller flap harvest: Technical insights from a case serie

**DOI:** 10.1016/j.ijscr.2025.111921

**Published:** 2025-09-08

**Authors:** Elise Lupon

**Affiliations:** a University Institute of Locomotor and Sport (IULS), Pasteur Hospital, 30 voie romaine, 06100, Nice, France; b Université Côte d'Azur, CNRS, LP2M, France

**Keywords:** Pedicled perforator flap, Intraoperative perforator identification, Propeller flap, Dorsal intercostal artery perforator (DICAP), Soft-tissue coverage, Reconstructive surgery

Dear Editor,

When preoperative perforator mapping is missing or unreliable, flap dissection can quickly become a challenge. An illustration of a simple intraoperative method to safely identify and harvest pedicled perforator flaps for back defects is presented here, following the SCARE guidelines [1]. Perforator propeller flaps are outstanding tools for optimized soft-tissue coverage, but they require a learning curve to master the dissection technique [2]. Preoperative perforator mapping, most often performed with a handheld Doppler, is the standard approach, followed by elevation of the flap in a distal-to-proximal fashion [3]. Duplex ultrasound now offers greater precision, but requires specific training [4]. In daily practice, acoustic Doppler mapping can be imprecise, misleading [5], or simply omitted, particularly in emergencies, when defects are unanticipated, or during late surgical decision-making.

In such situations, the perforator can be identified directly through the defect to secure the harvest. Under direct vision, the assistant gently elevates the surrounding skin with two skin hooks or a retractor such as a Farabeuf retractor, while the surgeon performs meticulous blunt dissection using fine, round-tipped scissors to avoid vessel injury (Fig. 1). Once skeletonized, designing the skin paddle becomes straightforward: the flap is tailored around the vessel, with incisions along its edges under direct visual control, a safe and reassuring step. After complete subcutaneous elevation on both sides of the perforator, the flap can be rotated more than 90° to achieve stable coverage.

**Fig. 1:**
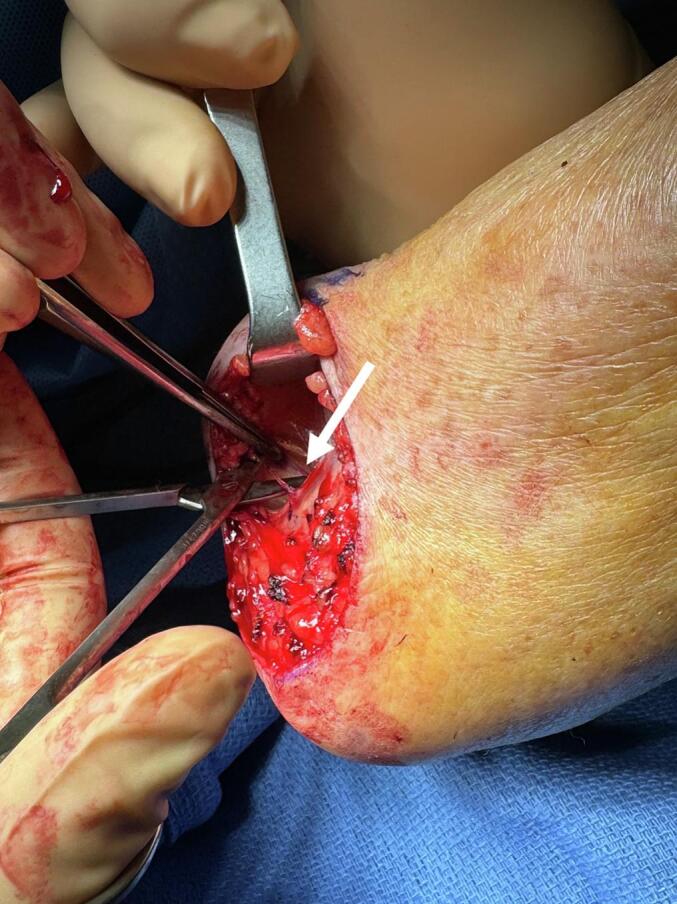
Tip for identifying a perforator adjacent to the defect. The assistant, positioned by the surgeon, gently elevates the skin to allow dissection from the wound edges. The surgeon dissects parallel to the perforator's course using short scissors. The white arrow indicates the appearance of the vessel when first suspected, before complete skeletonization. The assistant must maintain skin elevation throughout the dissection. Printed with permission; copyrights retained by Elise Lupon, MD, PhD.

Case 1 illustrates this rescue approach in an emergency setting. The reconstructive surgeon was called intraoperatively by the spine team to cover multiple exposed vertebrae after removal of infected osteosynthesis material in an elderly patient. A second anesthesia was to be avoided. Although the defect had not been anticipated, this technique enabled the rapid identification of two sizable intercostal perforators on either side of the defect, allowing prompt and reliable coverage using two DICAP flaps (Fig. 2).

**Fig. 2:**
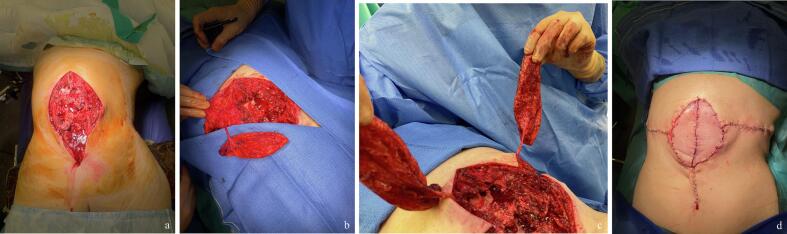
Case 1 *–* Double DICAP flaps designed from direct perforator identification through the defect in an emergency setting. a. Preoperative view of an extensive soft-tissue defect after debridement of devitalized and infected tissues. In a frail patient, immediate coverage was required to allow a one-stage procedure as requested by the anesthesia and spine teams. b. Dissection after identification of a dorsal intercostal perforator from the defect, achieved by gentle elevation of the wound edges. c. Elevation of two DICAP flaps oriented perpendicular to the defect axis, taking advantage of available skin laxity for direct donor-site closure. d. Final intraoperative view showing the defect fully covered with two DICAP flaps based on good-caliber perforators identified directly through the defect. Printed with permission; copyrights retained by Elise Lupon, MD, PhD.

Case 2 demonstrates this technique's usefulness in elective surgery. A patient with a basal cell carcinoma of the back underwent excision with minimal margins. Intraoperative assessment revealed that direct closure would result in excessive tension. Elevation of the adjacent skin exposed a good-caliber perforator, which served as the pivot for a small DICAP flap harvested along relaxed skin tension lines (Langer's lines). This redistributed tension away from the primary closure site and ensured durable coverage (Fig. 3).

**Fig. 3:**
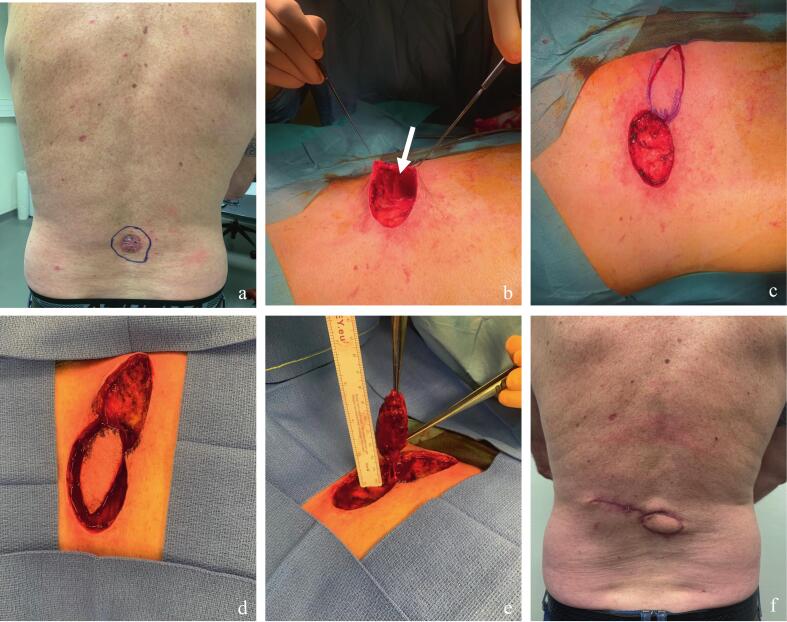
Case 2 - DICAP flap designed from direct perforator identification through the defect in an elective setting. a. Preoperative view of an extensive basal cell carcinoma (BCC) with marked surgical margins of 0.5 cm; direct closure was considered at high risk for postoperative dehiscence. b. Identification of a perforator (white arrow) by gentle elevation of the skin with two Gillies skin hooks held by the assistant. The surgeon is positioned opposite the elevated area for optimal visibility. c. Custom skin paddle design following Langer's lines of minimal tension over the flap donor site. d. Complete incision of the flap. e. Full skeletonization of the identified perforator, allowing rotation of approximately 160°. f. Five-weeks postoperative result showing good flap viability and absence of dehiscence, with redistribution of skin tension. Printed with permission; copyrights retained by Elise Lupon, MD, PhD.

This straightforward tip, particularly valuable for surgeons early in their perforator flap experience, can save significant operative time when Doppler mapping is uncertain or has failed [5]. It is especially relevant in cases of poor preoperative planning, emergencies involving unfamiliar teams or locations, or in resource-limited environments without sterile probes [6]. It is most suited to dorsal regions and other zones where subcutaneous fat is minimal and perforators can be easily dissected through the defect. Its main limitations are that only perforators adjacent to the defect can be identified, no suitable vessel may sometimes be found, and overly aggressive elevation can compromise the possibility of subsequently designing a keystone flap if needed. It is less applicable in very fatty areas such as the inguinal or axillary folds, and unsuitable for distant perforators, which in the context of pedicled flaps is rarely desirable anyway. While this tip is widely known among experienced flap surgeons, it is seldom well illustrated in the literature.

## Author contribution

**E. Lupon** conceived of the presented idea wrote the first draft of the manuscript, operate the patient and collected data, reviewed the final manuscript.

## Consent

All patients had signed informed consent for anonymous use of their data and for body donation to science while they were alive.

## Ethical approval

This study was performed in line with the principles of the Declaration of Helsinki. This study was approved by the Nice institution's Research Ethics Committee on August 14th 2025.

## Guarantor

Elise Lupon.

## Research registration number

1. Name of the registry: IORG0012275 - iULS-University Institute for Locomotion and Sport Case series

3. Hyperlink to your specific registration (must be publicly accessible and will be checked): N/A It is not the first in Man case report.

## SCARE guideline

The work has been reported in line with the SCARE criteria 2025.

## Funding

None. This research received no specific grant from any funding agency in the public, commercial, or not-for-profit sectors.

## Conflict of interest statement

The authors declare no funding and no conflict of interest related to this work.
